# Toll-like Receptor 7 Deficiency Attenuates Platelet Dysfunction in Sepsis

**DOI:** 10.3390/biom15111604

**Published:** 2025-11-15

**Authors:** Rashida Mohamed-Hinds, Arijit Dutta, Chanhee Park, Xiaomei Yang, Lin Zou, Wei Chao, Brittney Williams

**Affiliations:** Translational Research Program, Department of Anesthesiology, Center for Shock, Trauma and Anesthesiology Research, University of Maryland School of Medicine, Baltimore, MD 21201, USA

**Keywords:** sepsis, platelets, sepsis-induced platelet dysfunction, Toll-like receptor 7, inflammation, extracellular vesicles

## Abstract

Sepsis is a clinical syndrome caused by abnormal host response to infection. Thrombocytopenia and platelet dysfunction are common findings in sepsis and associated with worse outcomes. The innate immune single-stranded RNA sensor, Toll-like Receptor-7 (TLR7), plays a key role in thrombocytopenia in sepsis. This study investigated whether TLR7 signaling also contributes to platelet dysfunction in sepsis, and whether the bioactivity of downstream inflammatory mediators, specifically extracellular vesicles (EVs), is impacted by the TLR7 signaling pathway. Sepsis was induced in wild-type (WT) and TLR7-deficient (TLR7^−/−^) mice by cecal ligation and puncture. Blood was collected at twenty-four hours for platelet and plasma isolation, and platelet function was assessed using aggregation, adhesion, and calcium flux assays. EVs were isolated from plasma and used in vitro to evaluate their impact on platelet–leukocyte aggregate (PLA) formation. We found that septic platelets are highly activated and more adhesive, yet show markedly impaired aggregation and reduced calcium signaling, indicating functional exhaustion despite activation. Notably, mice lacking TLR7 maintained stronger platelet aggregation, enhanced adhesion, and preserved calcium release in the septic state compared to wild-type controls, suggesting a protective effect of TLR7 deficiency. Plasma EVs increased in abundance and size during sepsis and promoted clot and PLA formation in vitro. Notably, EV-mediated platelet activation was reduced with EVs derived from TLR7-deficient mice. Our results demonstrate that while sepsis drives persistent platelet activation and dysfunction, TLR7 deficiency preserves platelet function and modulates the pathogenic activity of EV-mediated platelet activation, highlighting TLR7 as a key regulator and potential therapeutic target in sepsis-induced platelet dysfunction.

## 1. Introduction

Sepsis is a severe clinical syndrome caused by a dysregulated host response to an infection and is characterized by excessive inflammation and multi-organ failure. Sepsis-induced coagulopathy (SIC) is a major complication of sepsis with an incidence of 24% in the United States that reaches up to 60% globally [1,2]. A hallmark of SIC is early and persistent thrombocytopenia, which occurs in up to 83.5% of patients, with 1 in 5 cases progressing to intravascular coagulation [3]. In sepsis, platelets are progressively activated, and consumed via endothelial sequestration, within platelet–leukocyte aggregates (PLA) and in microvascular thrombi. Additionally, research has shown impaired platelet aggregatory function in sepsis, which was associated with poor survival and served as an independent predictor for 28-day mortality [4].

Inflammation alters the platelet phenotype to promote interaction with endothelial and immune cells during infection and coincides with increased markers of platelet activation seen in septic patients [5,6,7], but these processes can simultaneously drive platelet dysfunction [8,9]. This has been observed in critically ill COVID-19 patients, where platelets show increase activation markers but are less responsive to external stimuli [10]. Sepsis triggers the release of damage-associated molecular patterns (DAMPs) from injured cells and tissues, which intensifies inflammation through innate immune receptor activation [2]. Persistent inflammatory signaling can result in ongoing platelet activation and eventual exhaustion, characterized by hyporesponsive platelets and impaired in vitro function.

Our previous findings have demonstrated the influence of innate immune receptors, specifically Toll-like receptors (TLRs), on sepsis pathology. Notably, TLR7 plays a critical role in ongoing platelet–leukocyte aggregate (PLA) formation [11] and development of thrombocytopenia [12] in septic mice. Further, we have shown that TLR7 genetic deficiency (TLR7^−/−^) in sepsis results in decrease inflammation, organ injury [13,14], and mortality [15]. TLR7 is an innate immune sensor for single-stranded RNA, including extracellular (ex) micro(mi)RNAs [16,17]. We found that circulating plasma extracellular RNA is increased in both septic mice and humans, the predominant biotype is miRNAs, and importantly, specific miRNAs induce significant cytokine production in macrophages acting through TLR7 [18]. These findings suggest that ex-miRNAs can act as innate immune activators via TLR7 in sepsis.

Ex-miRNA is carried in plasma by high-density lipoproteins, Argonaute (Ago)-2 protein, and extracellular vesicles (EVs), each of which protects them from plasma RNAse digestion [19]. EVs are nano-sized membrane bound particles that facilitate intercellular communications through the transfer of biological molecules and activate signaling pathways in recipient cells. In addition to miRNA/RNA, EVs carry other cargo such as proteins, lipids, and extracellular single- and double-stranded DNA, illustrating their potential functional diversity depending on host cell conditions [20]. For instance, septic plasma EVs induce marked cytokine production in macrophages [21] and in microglia [22] compared to sham/control EVs. Further, platelet-derived EVs from COVID-19 patients modulated NF-κB activation and IL-1β/TNFα/IL-8 upregulation, resulting in enhanced neutrophil extracellular trap (NET) formation [23]. Importantly, each of these studies demonstrated the impact of TLR7 in mediating pathological EV inflammatory signaling.

Other studies have highlighted the procoagulant potential of plasma EVs due to expression of tissue factor (TF) and phosphatidylserine [24]. For example, phosphatidylserine was increased on endothelial cells (ECs) treated with septic serum compared to healthy serum, and both septic serum and septic plasma EVs shortened clotting time and increased thrombin generation in cell samples [25]. In our most recent study, we demonstrated that platelets exposed to septic plasma exhibit elevated surface expression of the activation marker CD62p, and this response was significantly attenuated in TLR7^−/−^ platelets [11], suggesting that septic plasma contains a mediator(s) acting through TLR7, with evidence implicating septic EVs and associated cargo [21].

Based on the aforementioned reports, we hypothesize that TLR7 signaling impacts platelet function during sepsis, with septic EVs serving as circulating plasma mediators that facilitate platelet activation via TLR7. In this current study, we specifically present investigations into the impact of TLR7 on platelet aggregation and adhesion in sepsis, as well as the involvement of TLR7 in EV-mediated platelet activation. Our primary objective is to provide evidence for the role of TLR7 in modulating hemostatic platelet functions to identify a novel signaling pathway contributing to platelet dysfunction and coagulopathy in sepsis.

## 2. Materials and Methods

### 2.1. Animals

Experiments were performed using 10–24-week-old male WT (C57BL/6J, stock no. 000664) mice, and age/sex-matched TLR7^−/−^ (TLR7^tm1Flv^/J, stock no. 008380) mice, when applicable, purchased from the Jackson Laboratories (Bar Harbor, ME, USA). Mice were subsequently bred and housed in a temperature-controlled, pathogen-free environment with a 12-hour light–dark cycle and unlimited access to bacteria-free water and food within the animal facility at the University of Maryland School of Medicine (Baltimore, MD, USA). All animals were given a minimum of 7 days to acclimate to the animal facility prior to use in any experiments.

### 2.2. Polymicrobial Sepsis Model

We performed cecal ligation and puncture (CLP) procedure as previously performed [12] to induce polymicrobial abdominal sepsis. Briefly, mice were anesthetized with intraperitoneal (IP) injection of ketamine (120 mg/kg) and xylazine (4 mg/kg), and CLP was performed via laparotomy. Subcutaneous buprenorphine (0.1 mg/kg) was given preemptively for pain management. Cecal ligation was performed 1.5 cm from the distal tip of the cecum and a through and through puncture with an 18-gauge needle was made mid cecum and 2–3 mm of feces was extruded. The cecum was then placed back into the peritoneal cavity and the peritoneum and skin closed. Prewarmed normal saline (0.3 mL/g) was administered subcutaneously (SQ) at the end of each procedure for fluid resuscitation, and bupivacaine (3 mg/kgh) was administered SQ for additional postoperative pain control. Sham mice underwent the same procedures, including the laparotomy, but without CLP. Four or twenty-four hours after sham or CLP surgery, rectal temperature was taken and recorded.

### 2.3. Blood Collection

Blood samples were collected 4 or 24 h after surgery via euthanization with IP administration of ketamine/xylazine as above followed by exsanguination via cardiac puncture. Blood was transferred to microcentrifuge tubes containing 3.2% sodium citrate maintaining a 1:8 anticoagulant/blood ratio, and then centrifuged for the isolation of platelets, platelet-rich plasma (PRP), or the collection of platelet-poor plasma for EV isolation and future experiments. Platelet counts were measured by an automated cell counter using the Coulter Ac●T diff2 Hematology Analyzer (Beckman Coulter, Indianapolis, IN, USA), or the Nexcelom Cellometer K2 ((Revvity, Waltham, MA, USA) for select experiments.

### 2.4. Platelet Isolation

Washed platelets were prepared as previously described to maximize platelet yield and minimize leukocyte contamination [11]. Briefly, anticoagulated blood was mixed gently with 400 µL of Tyrode’s buffer (134 mM NaCl, 12 mM NaHCO_3_, 2.9 mM KCl, 0.34 Na_2_HPO_4_, 10 mM HEPES, 1 mM MgCl_2_, 5 mM Dextrose, and 3 mg/mL of bovine serum albumin (BSA)) and centrifuged at room temperature for 8 min at 50× *g*. The supernatant plus the top layer of red blood cells (approximately 600 µL) were transferred into new microcentrifuge tubes and centrifuged at 100× *g* for 5 min. Without touching the buffy coat or the sedimented red cell layer, the platelet-rich plasma (PRP) layer was collected and pooled from two to four mice into a new microcentrifuge tube and concentrations adjusted to 0.5–1 × 10^5^ platelets/μL for downstream experiments. A third centrifugation step was performed on the PRP at 800× *g* for 8 min to obtain washed platelets. The supernatant was removed, and the platelet pellet was washed gently once with CGS-EDTA buffer (100 mM NaCl, 8.5 mM Tris, 8.5 mM Dextrose, and 1 mM EDTA). Platelets were then resuspended to concentrations of 1–1.5 × 10^5^ platelets/μL in Tyrode’s buffer (TB) for downstream experiments.

### 2.5. Platelet Aggregation Assay

Platelet aggregation was monitored and recorded using light transmission aggregometry on a PAP-8E platelet aggregometer (Biodata, Horsham, PA, USA). Briefly, 225 µL of resuspended platelets in TB w/o calcium at concentration of 1.5 × 10^5^ platelets/μL were stimulated with thrombin 0.1 u/mL (T1063, Sigma Aldrich, St. Louis, MO, USA) or ADP 20 μm (101312, BioData, Horsham, PA, USA). Platelets were monitored for aggregation under continuous stirring for 6 min at 1200 rpm at 37 °C by measuring changes in light transmission compared to a ‘blank’ sample (100% light transmission). Data are presented as averages of the primary slope (or rate- % aggregation(agg)/min) and % maximal aggregation across all experimental sets.

### 2.6. Platelet Adhesion Assay

Platelet adhesion was measured using a microfluid syringe pump (Cellix, ExiGo, Dublin, Ireland). Vena8 Fluoro+ Biochips (biochip w/8 parallel capillaries, Cellix, Dublin, Ireland) were coated with soluble collagen (rat collagen type 1, Sigma Aldrich, C7661) at 200 µg/mL and left overnight at 4 °C. This was followed by surface blocking with denatured bovine serum albumin (BSA) for 30 min at room temperature. Biochips were then washed with PBS buffer prior to experimental use. Using the ExiGo pump, 120 μL of washed platelets (1 × 10^5^ /μL) in TB w/calcium was perfused across collagen-coated capillaries at a shear rate of 1000 s^−1^ (40 µL/min). At the end of perfusion, the channels were washed once at the same rate with PBS to remove any non-adherent cells and then imaged at 10X magnification with a Nikon Eclipse Ti2-E Inverted Microscope (Nikon, Japan). Attached platelets were imaged at three prelabeled points on each capillary (2−4−6) representing the beginning–middle–end fields of the channel, across all capillaries. Data from each field was analyzed using ImageJ software (v1.54d) with the desired objects (platelets) coded as red and is expressed as the total number of adhered platelets and the total coverage area (µM per channel) and then averaged for each experimental group.

### 2.7. Platelet Factor-4 Secretion

Platelet-poor plasma from WT and TLR7^−/−^ sham and CLP mice were processed from whole blood following a three-step centrifugation process (1000× *g* twice and 10,000× *g* once). The plasma was assayed for platelet factor-4 (PF4) using an R&D mouse CXCL4/PF4 DuoSet ELISA (DY595, Minneapolis, MN, USA)) and following the manufacturer’s protocol. Data is expressed as pg/mL based on generation of a standard curve from known PF4 concentrations and 4PL analysis.

### 2.8. Cytosolic Ca^2+^ Transients in Platelets

Pooled PRP was loaded with FURA-2 AM (Invitrogen F1221, Carlsbad, CA, USA) at 5 μm for 60 min at 37 °C under constant rotation. Following washing and centrifugation, the platelet pellet was resuspended in TB w/o calcium (0.5–1 × 10^5^/μL) and changes in cytosolic calcium were measured using a SpectraMax M5 plate reader (Molecular Devices, San Jose, CA, USA). Platelets were added to a cuvette, and after 60 s, thrombin (0.1 or 1 u/mL) was added to the samples. Intracellular calcium [Ca^2+^]i concentration was determined by measuring FURA-2 AM fluorescence at 510 nm, using 340/380 nm dual-wavelength excitation for 5 min with 7 s interval recordings. In each sample, the first 60 s of recording was used to obtain a baseline prior to measurement, and the total acquisition time was 6 min and 45 s. All measurements occurred at 37 °C. Calibration was performed at the end of each experiment by the addition of 0.1% TritonX-100 for the maximum fluorescence ratio and then 6 mM EGTA for the minimum fluorescence ratio. The total [Ca^2+^]i released over time (or AUC) was calculated from the 340/380 nm excitation fluorescence ratios [26] using a Ca-FURA-2 AM dissociation constant (K_d_) in platelets of 224 nM, as described previously for platelets [27]. Per the manufacturer, by using the ratio of fluorescence intensities produced by excitation at these two wavelengths, factors such as uneven dye distribution and leakage are minimized because they will affect both measurements to the same extent. Finally, we alternated the measurement of samples between the different groups to minimize the impact of the incubation time prior to fluorescent measurements.

### 2.9. Ex Vivo Impact of CLP Plasma on Platelet Activation

For select experiments, washed platelets (1 × 10^5^/μL) isolated from naïve mice were incubated with pooled plasma from WT or TLR7^−/−^ sham and CLP mice. For platelet activation, 20% *v*/*v* of plasma was added to naïve platelets and incubated at 37 °C for 15 min under constant rotation. Platelets were stained with PE-anti-CD41 (BD Biosciences PMG-553848; Clone MWReg30, Minneapolis, MN, USA), constitutive platelet marker, and AF647-anti-CD62p (BD Biosciences PMG-563674; Clone Rb40.34, Minneapolis, MN, USA) platelet activation marker, and then treated with 2% paraformaldehyde in a 1:1 *v*/*v* ratio for fixation. Samples were centrifuged, supernatant removed, and the cell pellet resuspended in FACS buffer (DPBS/5%FBS). Data was recorded on the BD LSR II Flow Cytometer and analyzed with FlowJo version(V) 10.10 software. The activation status of platelets was determined by increased expression of CD62p. (CD41^+^/CD62p^+^). The gating strategy is shown in Supplementary Materials Figure S1.

### 2.10. Plasma EV Isolation and Quantification

EVs were isolated from the plasma of WT or TLR7^−/−^ sham and CLP mice by ultracentrifugation, as we described previously [22]. In brief, EDTA-anticoagulated pooled plasma (n = 2–4 mice) was prepared by centrifugation, diluted 1:1 with sterile, cold DPBS, and then centrifuged at 12,000× *g* at 4 °C for 30 min. The supernatant was removed and transferred to a polycarbonate ultracentrifuge tube, and the total volume was brought up to 5 mL using sterile, cold DPBS and aseptic technique. Samples were subjected to ultracentrifugation at 110,000× *g* at 4 °C for 1 h using the Optima MAX-XP ultracentrifuge with MLA-80 rotor (Beckmann Coulter, Indianapolis, IN, USA). After centrifugation, the supernatant was discarded, leaving approximately 150 µL of solution. The pellet containing EVs was then resuspended in the remaining solution in the tube. EV size and concentration were quantified using the ViewSizer^®^ 3000 Nanoparticle Tracking Analysis (NTA) system (Horiba Instruments, Irvine, CA, USA), aliquoted, and stored at −80 °C until future use.

### 2.11. Plasma EV Treatment In Vitro

#### 2.11.1. Hemostatic Viscoelastic Testing

Whole blood from naïve mice were incubated with sham and CLP plasma EVs for coagulation testing. Briefly, 180 µL of whole blood was incubated with 20 µL of EVs at a concentration of 3 × 10^10^ EV/mL. A ROTEM NaTEM assay (star-tem 000503-10-US, Werfen, Bedford, MA, USA) was performed by the re-calcification of whole blood samples. Time to 2 mm fibrin polymerization, or the clotting time (CT), and the maximum clot firmness (MCF) were measured and recorded using ROTEM Delta platform (Werfen, Bedford, MA, USA). CT and MCF are indirect measures of thrombin generation, representing speed and strength of clot formation, respectively. Sample measurements were run at 37 °C for 30 min. For each set, fresh whole blood was collected in order to minimize the impact of time prior to recalcification and ROTEM measurements.

#### 2.11.2. Flow Cytometry

Whole blood from WT or TLR7^−/−^-naïve mice were incubated with sham or CLP plasma EVs from WT or TLR7^−/−^ donor mice, and quantification of activated platelet–leukocyte aggregates (PLAs) performed. Ninety microliters of 3.2% citrate-anticoagulated blood were incubated with 10 uL of plasma EVs at a concentration of 2.5 × 10^10^ EV/mL for 30 min. Blood was then stained with PerCP CY5.5-anti-CD45 (BioLegend BLD-103131; Clone 30-F11, San Diego, CA, USA)) pan-leukocyte marker, PE-anti-CD41 platelet marker, and AF647-anti-CD62p platelet activation marker for 15 min. Samples were then treated with BD Phosflow^™^ Lyse/Fix Buffer (Franklin Lakes, NJ, USA) for 10 min, followed by washing and centrifugation. The cell pellet was resuspended in 450 uL of FACS buffer. Data was recorded using the BD Aurora Cytek flow cytometer (Bethesda, MD, USA) and analyzed using FlowJo V10.10 software. PLAs were determined based on forward (FSC) and side (SSC) scatter properties and double positive expression of CD41 and CD45, and the activation status of platelets within PLA was determined by increased expression of CD62p (CD45^+^/CD62p^+^). The gating strategy is shown in Supplementary Materials Figure S2.

### 2.12. Group Assignment and Statistical Analysis

Mice were randomly assigned to sham or CLP groups, and operators were blinded to strain and group information for experiments until after statistical analysis and quality data checks were complete. GraphPad Prism 10 software was used for all statistical analysis (La Jolla, CA, USA). Continuous variables were expressed as mean ± SD. Data was tested for Gaussian (normal) distribution using the D’Agostino & Pearson test, and parametric or non-parametric tests were applied accordingly. For comparison between two groups, unpaired Student’s *t*-tests or Mann–Whitney tests were used according to data distribution. For comparison among more than two groups, statistical significance was determined by one-way ANOVA with post hoc Dunnet’s (comparing against control group) or Bonferroni’s test for multiple comparison if data showed normal distribution and non-significant differences in SDs (variance) based on the Brown–Forsythe test. If these criteria were not met, then Brown–Forsythe and Welch ANOVA were used for multiple comparisons. Data is presented as mean ± SD. A two-tailed *p* value < 0.05 was considered statistically significant.

## 3. Results

### 3.1. Platelet Function Is Altered in Mice with Cecal Ligation and Puncture-Induced Sepsis

**Figure 1:**
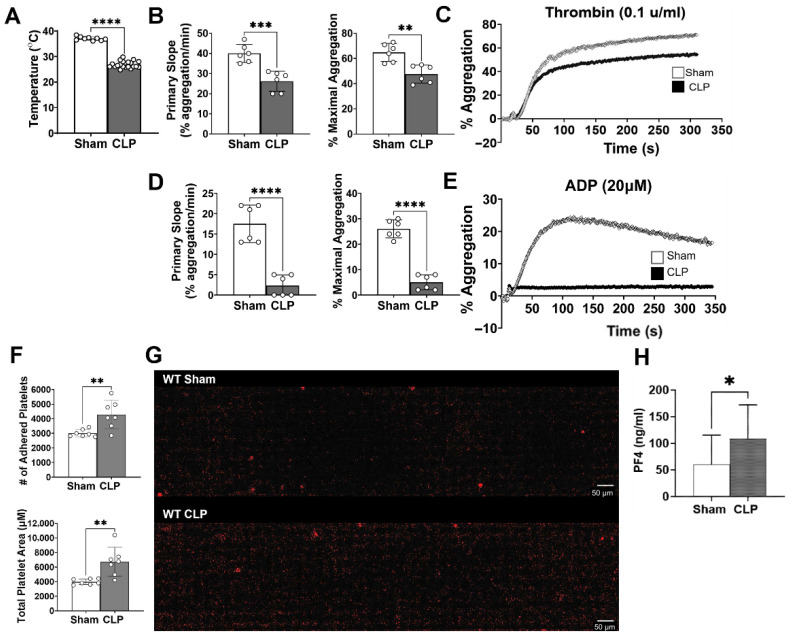
Platelet function is altered in mice with cecal ligation and puncture-induced sepsis. Wild-type mice underwent sham or CLP procedures. Blood was collected and platelet isolates combined from two sham and three to four CLP mice per set for a total of two to three sets. At 24H, average temperatures for CLP sets were significantly lower than sham (**A**). Isolated platelets were combined and resuspended to equal concentrations (1–1.5 × 10^5^ platelets/µL) across all sets for aggregometry and adhesion studies. CLP platelets showed a significantly reduced rate of aggregation (primary slope) and maximal aggregation in response to thrombin (0.1 u/mL) (**B**) and ADP (20 µM) (**D**) compared to sham; representative images are shown for thrombin (**C**) and ADP-induced aggregation (**E**). n = 6 samples, three sets ran in duplicate for thrombin, and two sets ran in triplicate for ADP. Isolated platelets from sham or CLP mice were perfused over collagen (200 µg/mL)-coated microfluidic channels at a shear rate of 1000 s^−1^ (40 µL/min). Adhered number of platelets and surface coverage area (µm) was determined. CLP platelets show an increased number of adhered platelets and platelet coverage area compared to sham (**F**); representative images are shown for sham and CLP. Scale bar 50 µm. (**G**). n = 7 samples, two sets ran in triplicate and quadruplicate. Finally, at 4H, plasma levels of PF4, a marker of platelet activation, are increased in CLP mice versus sham (**H**). *p* < 0.05 *, *p* < 0.01 **, *p* < 0.001 ***, *p* < 0.0001 ****.

To characterize the impact of a septic state on essential platelet functions, we evaluated platelet aggregation, adhesion, and activation. At 24 h, CLP mice had notable hypothermia compared to sham mice (37 ± 0.73 °C vs. 26.9 ± 1.3 °C, *p* < 0.0001) (Figure 1A) indicating the severity of septic shock in our CLP model. In response to thrombin, we observed a 34.6% and 26.5% reduction in the rate of, and % maximum, platelet aggregation, respectively, in CLP mice compared to sham (Figure 1B,C). Stimulation with ADP demonstrated further reductions in the rate of aggregation (17.5 ± 4.6% agg/min vs. 2.3 ± 2.4% agg/min, *p* < 0.0001) and maximal aggregation (26 ± 3.5% max agg vs. 5 ± 3% max agg, *p* < 0.0001) in CLP mice compared to sham (Figure 1D,E). Based on the premise that septic platelets circulate in an activated state [28], we next evaluated whether this impacted platelet adhesion function. To simulate microvascular flow, we used a microfluidic device to perfuse isolated platelets across collagen-coated micro channels. While both sham and CLP platelets adhered to the channels, CLP platelets showed a significant increase in the total number of adhered platelets (3009 ± 249.1 platelets vs. 4289 ± 968.4 platelets, *p* = 0.0054) and the total coverage area (3989 ± 393.7 µM vs. 6740 ± 1981 µM, *p* = 0.0036) (Figure 1F,G). Further, four hours after CLP, we noted that septic mice had twice the plasma levels of soluble PF4 compared to sham (60.1 ± 55.3 pg/mL vs. 108.2 ± 64 pg/mL, *p <* 0.05) (Figure 1H), indicating an increase in systemic platelet activation. These findings are consistent with our prior work in which platelets from septic mice expressed higher amounts of platelet activation marker CD62p compared to those of sham mice early in the disease course [11]. Collectively, these results demonstrate that platelet function is not uniformly impacted by the septic state; platelet adhesion is maintained, and even augmented in septic platelets, while aggregatory ability is severely impaired in response to exogenous agonists.

### 3.2. TLR7-Deficient Mice Have Improved Platelet Aggregation and Enhanced Platelet Adhesion in Sepsis

**Figure 2:**
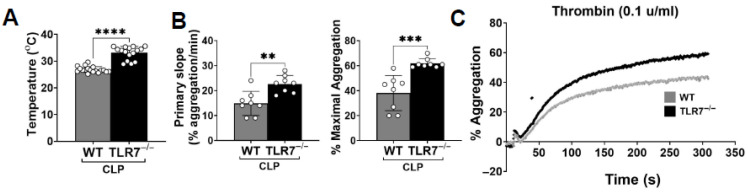

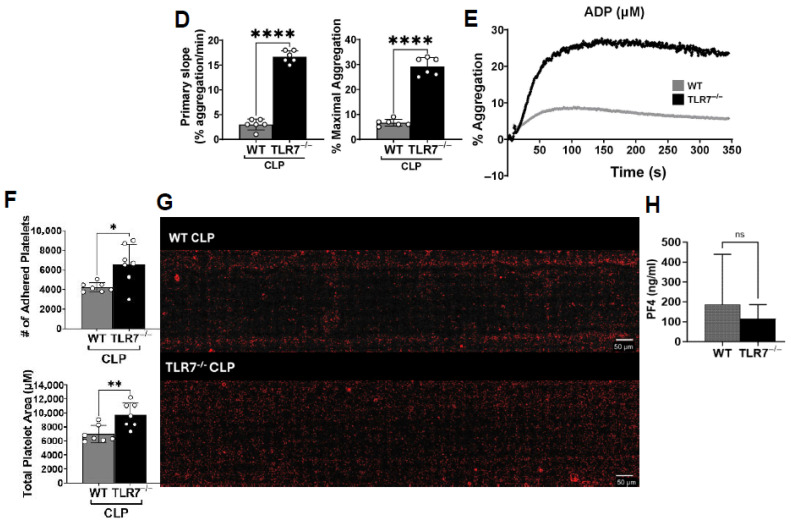
TLR7 deficiency improves both platelet aggregation and platelet adhesion in sepsis. Wild-type and age/sex matched TLR7^−/−^ mice underwent a CLP procedure. Blood was collected and platelet isolates combined from three to four CLP mice per set for a total of two to three sets. At 24H, average temperatures for WT CLP sets were lower than TLR7^−/−^ sets (**A**). Isolated platelets were combined and resuspended to equal concentrations (1–1.5 × 10^5^ platelets/µL) across all sets for light transmission aggregometry and platelet adhesion studies. WT CLP platelets showed a significantly reduced rate of aggregation (primary slope) and maximal aggregation in response to thrombin (0.1 u/mL) (**B**) and ADP (20 µM) (**D**) compared to TLR7^−/−^ CLP platelets; representative images are shown for thrombin (**C**) and ADP-induced aggregation (**E**). n = 8 samples, three sets ran in duplicate or triplicate for thrombin, and n = 6 samples, two sets ran in triplicate for ADP. Isolated platelets from WT and TLR7^−/−^ CLP mice 24H post procedure were perfused over collagen (200 ug/mL)-coated microfluidic channels at a shear rate of 1000 s^−1^ (40 µL/min). Adhered number of platelets and surface coverage area (µM) was determined and analyzed using ImageJ software. TLR7^−/−^ CLP platelets show an increased number of adhered platelets and platelet coverage area compared to WT CLP platelets (**F**); representative images are shown for WT and TLR7^−/−^ mouse platelets. Scale bar 50 µm (**G**). n = 7 samples, two sets ran in triplicate and quadruplicate. There was no difference in plasma PF4 levels between WT and TLR7^−/−^ mice at 4H (**H**). ns = not significant, *p* < 0.05 *, *p* < 0.01 **, *p* < 0.001 ***, *p* < 0.0001 ****.

Our prior work demonstrates that TLR7 deficiency results in a protective effect against sepsis-induced thrombocytopenia [11,12]. To determine whether this extends to platelet function, we isolated platelets from TLR7^−/−^ sham and CLP mice and evaluated both adhesive and aggregatory function compared to WT mice. It is noted that the aggregatory response of platelets from sham WT and TLR7^−/−^ mice showed no difference in response to thrombin and ADP (Supplementary Materials Figure S3A,B) and showed no difference in the number (sham WT 3608 platelets vs. sham TLR7^−/−^ 3162 platelets) or area (sham WT 4411 µM vs. sham TLR7^−/−^ 4683 µM) of adhered platelets to collagen-coated channels, indicating no baseline differences in platelet function for WT and TLR7^−/−^ sham mice. When compared with WT mice, TLR7^−/−^ mice had moderately higher temperatures 24 h after CLP (26.8 ±1.1 °C vs. 33.1 ± 0.2.4 °C, *p* < 0.0001) (Figure 2A). Also, TLR7^−/−^ CLP mice demonstrate improved aggregatory function in response to thrombin (Figure 2B,C) and ADP (Figure 2D,E) compared to WT CLP mice. Further, TLR7^−/−^ mice show higher platelet adherence to immobilized collagen in terms of total number (4234 ± 461.8 platelets vs. 6589 ± 2048 platelets, *p* = 0.0117) and coverage area (6979 ± 1215 µM vs. 9719 ± 1781 µM, *p* = 0.0057) (Figure 2F,G) of platelets. Finally, there was no difference in soluble PF4 levels between WT and TLR7^−/−^ CLP mice (185.8 ± 253.6 pg/mL vs. 115 ± 71.43 pg/mL, *p* = 0.2583) (Figure 2H). These results imply that TLR7 deficiency overall preserves, and even augments, platelet function in the setting of ongoing platelet activation in sepsis.

### 3.3. Septic Platelets Demonstrate Reduce Calcium Flux in Response to Thrombin Suggestive of Receptor Desensitization, While TLR7 Deficiency Preserves Calcium Response

**Figure 3:**
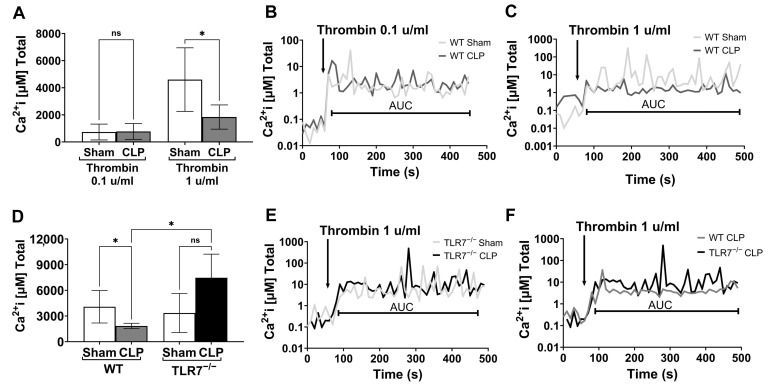
Septic platelets demonstrate reduce calcium flux in response to thrombin, suggestive of receptor desensitization, while TLR7 deficiency preserves calcium release. Blood was collected 24H post-procedure, and PRP samples were combined from sham (one to two mice) and CLP (two to three mice) groups for five sets. Platelet counts were equalized to 0.5–1 × 10^5^ platelets/μL in all PRP samples. PRP was incubated with FURA-2 AM (5 μm) in calcium- and magnesium-free Tyrode’s buffer at 37 °C with rotation for 60 min. Thrombin (0.1 or 1 u/mL) was added after 60 s, and fluorescence emission of 510 measured at dual excitation wavelengths, 340 and 380 nm. At low-dose thrombin (0.1 u/mL), sham and CLP showed similar increase in intracellular calcium transients ([Ca^2+^]i transients), while at a higher dose of thrombin (1 u/mL), CLP platelets display significant attenuation in [Ca^2+^]i transients and lower total intracellular calcium release (**A**); with representative images for low- and high-dose thrombin shown (**B**,**C**). n = 2 for low-dose thrombin and 6 for high-dose thrombin. To determine the impact of TLR7^−/−^ on platelet calcium transients, we utilized PRP from sham and CLP age/sex matched WT and TLR7^−/−^ mice. Platelet number was equalized between 0.5 and 0.75 × 10^5^ platelets/µL for WT and TLR7^−/−^ mice and incubated with FURA-2 AM and stimulated with thrombin (1 u/mL) as above. TLR7^−/−^ mice showed similar [Ca^2+^]i transients and total intracellular calcium release between sham and CLP (**D**,**E**). However, compared to WT CLP mice, TLR7^−/−^ CLP mice showed higher total [Ca^2+^]I release (**D**,**F**). n = 4–8 samples per group. ns = non-significant, *p* < 0.05 *.

Upon platelet receptor stimulation, calcium is released from the dense tubular system, initiating platelet aggregation through cytoskeletal changes, granule release, and fibrinogen receptor activation. Platelet calcium levels rise in rhythmic oscillations—initial spikes reflect direct stimulation, while subsequent spikes occur as neighboring platelets activate and propagate the signal, and it is this oscillatory flux that promotes efficient platelet aggregation [29]. We assessed platelet calcium transients in response to an escalating dose of thrombin (0.1 u/mL vs. 1 u/mL) in sham and CLP WT platelets to determine if the hyporesponsiveness observed is related to a reduction in oscillatory calcium release. Sham and CLP platelets demonstrate no difference in total calcium release in response to a lower dose of thrombin (730 ± 584 µM vs. 773 ± 584 µM, *p* = 0.9484), but at a higher dose, total calcium release in sham platelets was significantly greater than CLP platelets (4599 ± 2346 µM vs. 1838 ± 895 µM, *p* = 0.0336) (Figure 3A). We noted in the representative tracings that the initial response to exogenous stimulation is similar between WT sham and CLP platelets (Figure 3B,C), but positive oscillatory signals appear to decrease over time in CLP at the higher thrombin dose (Figure 3C), consistent with overall reduction in total intracellular calcium release. We next determined the impact of TLR7 deficiency on the blunted calcium flux we observed in CLP platelets. As shown in Figure 3D–F, while WT CLP platelets again show reduced total calcium release in response to high-dose thrombin compared to sham platelets (4080 ± 1901 µM vs. 1840 ± 310 µM, *p* = 0.0124), TLR7^−/−^ CLP platelets show significantly higher calcium flux compared to TLR7^−/−^ sham (3350 ± 2285 µM vs. 7469 ± 2760 µM, *p* = 0.0336) and WT CLP mice (*p* = 0.0260). These findings indicate that the lower calcium response in WT septic platelets is reversed with TLR7^−/−^ deficiency. Of note, we found no difference in baseline calcium flux between WT and TLR7^−/−^-naïve platelets (Supplementary Materials Figure S4A,B).

### 3.4. Effect of Septic EVs on Global Coagulation and Platelet Activation

**Figure 4:**
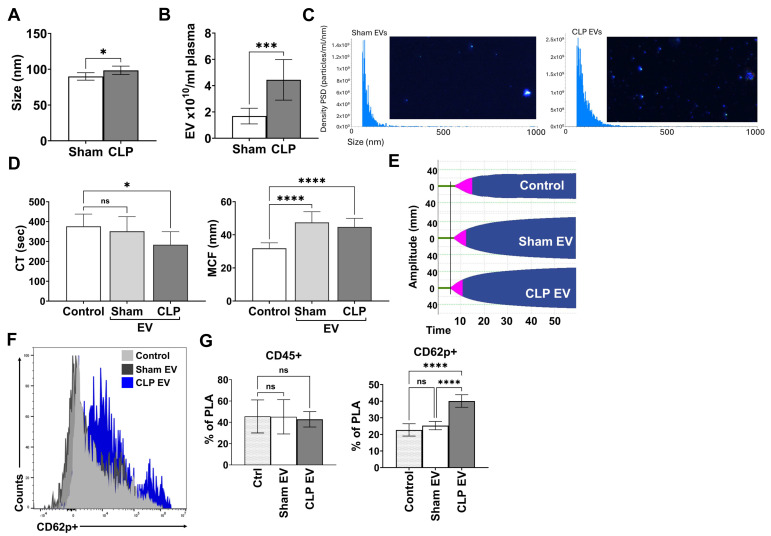
Functional characterization of EVs isolated from the plasma of sham and CLP mice. Mice were subjected to sham or CLP procedures. At 24H, whole blood was collected and EVs purified from processed plasma. Size was similar between sham and CLP plasma EVs (**A**), but CLP mice had higher plasma concentrations of EVs (**B**,**C**), as quantified by NTA ViewSizer® 3000. n = 7 sham and 8 CLP mice per group. Whole blood collected from naïve mice were incubated with sham or CLP EVs at a dose of 3 × 10^10^ EVs/mL for 1 min prior to recalcification of whole blood and assessment of global coagulation via ROTEM-NATEM. While CLP EVs shortened the CT compared to control, there was no difference between sham and CLP EVs with CT or maximum amplitude at 30 min (**D**); representative temograms shown (**E**). n = 8–12 samples per group. Whole blood from naïve mice was incubated with sham or CLP EVs and activated PLAs quantified (CD62p^+^CD45^+^/CD45^+^ PLA × 100%). Tyrode’s buffer was used as a control. Representative histogram (**F**) and bar graph (**G**) showing CLP EVs induce a significant increase in activated PLA formation in whole blood compared to control and sham samples. n = 5 samples per group. ns = not significant, *p* < 0.05 *, *p* < 0.001 ***, *p* < 0.0001 ****.

We have reported that plasma EVs and TLR7 play a role in systemic inflammation in polymicrobial sepsis [15,22]. In this study, we aimed to determine if septic plasma EVs could induce platelet activation and whether TLR7 deficiency impacts the response. We isolated EVs from sham and CLP mice as previously described [21,22], and determined their size and concentration using ViewSizer^®^ 3000 NTA (Horiba Instruments, Irvine, CA, USA). We found that plasma EVs were increased 2-fold in the plasma of septic mice compared to sham and slightly larger in size (Figure 4A–C). For subsequent experiments, EVs from sham and CLP mice were pooled and concentrations equalized for in vitro testing. To determine if septic plasma EVs mediate global coagulation, we utilized the viscoelastic platform ROTEM. Specifically, we utilized the NaTEM test, which re-calcifies the blood sample without the addition of exogenous coagulant activators and is sensitive to levels of endogenous tissue factor [30]. Septic EVs shortened the clotting time by 20% and sham EVs by 10% when compared to PBS controls, and both sham and septic EVs were able to augment maximum clot firmness (Figure 4D,E). Next, we determined whether septic EVs could induce platelet activation and PLA formation (CD45^+^/CD62p^+^) in whole blood. Septic plasma EVs significantly increase activated PLA formation compared to controls (22.7 ± 3.7 vs. 40.1 ± 3.8, *p* < 0.0001) and sham EVs (25.3 ± 2.5 vs. 40.1 ± 3.8, *p* < 0.0001) (Figure 4F,G).

### 3.5. TLR7 Signaling Impacts EV-Mediated Platelet Activation and PLA Formation in Sepsis

**Figure 5:**
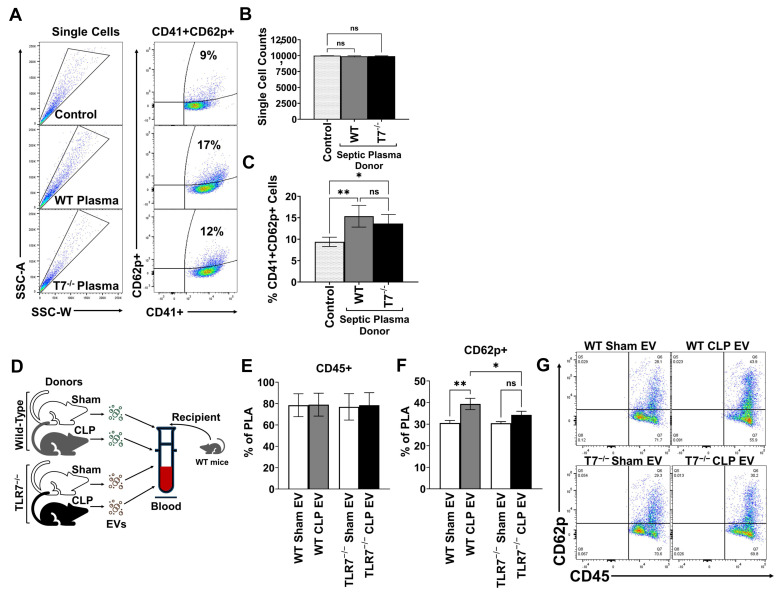
TLR7 signaling impacts EV-mediated platelet activation and PLA formation in sepsis. WT and age/sex matched TLR7^−/−^ mice underwent sham or CLP procedures; n= 3 mice per group. At 24H, blood was collected, processed to plasma, and combined for platelet activation experiments. Platelets were isolated from WT naive mice. Twenty percent *v*/*v* of WT or TLR7^−/−^ CLP plasma was added to the naïve platelet suspension. Platelet activation was defined as the percentage of CD62p^+^ platelets over total CD41^+^ platelets from single cells (CD62P^+^CD41^+^/CD41^+^ × 100%). Representative flow cytometry plots for WT and TLR7^−/−^ plasma treatments to naïve platelets (**A**) and corresponding bar graphs demonstrating a non-significant difference in platelet activation between WT and TLR7^−/−^ plasma treatments (**B**,**C**). n = 8 samples per group. EVs were purified from WT and TLR7^−/−^ sham and CLP plasma at 24H and incubated with whole blood from WT naïve mice (**D**) and activated PLAs were quantified. WT CLP EVs induced a significant increase in CD62p^+^ PLA compared to EVs derived from WT sham and TLR7^−/−^ CLP mice (**E**,**F**); representative flow plots shown for WT and TLR7^−/−^ sham and CLP EV treatments (**G**). n = 6 samples per group. ns = not significant, *p* < 0.05 *, *p* < 0.01 **.

We hypothesize that EVs signal in part via TLR7 to induce platelet activation in sepsis. We collected WT and TLR7^−/−^ whole blood from naïve mice, incubated with EVs from WT CLP mice, and quantified PLA formation as above. We found that WT CLP EVs induced similar PLA formation in both WT and TLR7^−/−^ blood samples (Supplementary Materials Figure S5). This implies that PLA formation is not dependent on EV→TLR7 signaling, and there are likely multiple signaling pathways involved. We then tested an alternative hypothesis that upstream TLR7 signaling in sepsis impacts downstream plasma EV-mediated platelet activation and PLA formation. We first determined potential differences in WT and TLR7^−/−^ septic plasma on platelet activation. We performed a transfer experiment in which WT platelets from naïve mice were incubated with donor WT or TLR7^−/−^ septic plasma and CD62p expression measured by flow cytometry. We observed that WT plasma significantly increased CD62p expression in platelets (CD41^+^/CD62p^+^) above control samples (Figure 5A–C). Expectedly, TLR7^−/−^ septic plasma also induced platelet activation; although the response was somewhat attenuated, it was not significantly different compared to CLP plasma treatment (15.36 ± 2.5% (WT) vs. 13.6 ± 2.1% (TLR7^−/−^) (Figure 5A–C). Next, we narrowed our focus and tested the impact of TLR7 on plasma EVs as a downstream effector in sepsis. EV-mediated platelet activation was measured by incubating whole blood from WT mice with plasma EVs from WT and TLR7^−/−^ sham and CLP mice (Figure 5D). WT CLP EVs induced significantly higher activated PLA formation compared to both WT sham EVs (30.6 ± 2.2% vs. 39.4 ± 2.6%, *p* = 0.0011) and TLR7^−/−^ CLP EVs (vs. 34.3 ± 4.7%, *p* < 0.0199) (Figure 5E–G). There was no difference in activated PLA formation between TLR7^−/−^ sham and TLR7^−/−^ CLP EV treatments (*p* = 0.2067). Therefore, EVs derived from TLR7^−/−^ septic mice exhibit a reduced capacity to promote PLA formation in comparison to EVs from WT septic mice. These results indicate that upstream TLR7 signaling can modulate the functional properties of septic plasma EVs.

## 4. Discussion

In the current study, we demonstrate that sepsis induces a distinct platelet phenotype characterized by increased platelet activation and adhesion (procoagulant), but also hyporesponsiveness, as evidenced by significantly reduced calcium transients and platelet–platelet aggregation. Notably, we demonstrate the contribution of TLR7, a single-stranded RNA sensor, to the development of procoagulant, but also to hypofunctional platelets, in a bacterial sepsis model. TLR7 deficiency led to a similar degree of platelet activation, but improved platelet aggregation, augmented adhesion, and preserved intracellular calcium responses. Extracellular vesicles (EVs) are recognized as damage-associated molecular patterns (DAMPs) which can signal through Toll-like receptor 7 (TLR7) to mediate inflammatory responses [21,22]. Although we did not observe that EV-mediated platelet activation was dependent on TLR7, we did find that plasma EVs appear to be downstream effectors of TLR7 inflammatory signaling, resulting in altered EV biological functions, specifically in terms of platelet activation and PLA formation.

Our findings are consistent with other sepsis studies demonstrating increased platelet activation [7] but reduced aggregation of platelets [31]. In our current study, exposure to collagen under shear stress resulted in an increased number of adhered septic platelets compared to sham. We postulate that the increased adherence of septic platelets may be secondary to their activated state, rendering them “stickier”. Pretreated platelets were shown to bind collagen to a higher degree compared to platelets without exposure to agonists [32]. Further, platelet GPVI is considered a primary receptor for collagen binding, but upon activation, platelet integrin receptor α_2_β_1_’s affinity for collagen increases, resulting in stronger adhesion to collagen [33]. These authors further demonstrate that in terms of binding to immobilized collagen, even with a blockade of the fibrinogen receptor needed for platelet crosslinking and aggregation, platelet adhesion is not impacted, supporting our findings that septic platelets can have preserved and augmented adhesive ability, but simultaneous reduction in aggregatory responses. A major finding of our study is the impact of TLR7 signaling on the development of platelet dysfunction in sepsis. Previously, we observed a role for TLR7 in the development of septic coagulopathy, in which TLR7 deficiency lead to reduce plasma levels of tissue factor (an initiator of coagulation) and cytokine IL-6, preservation of platelet counts, and better ex vivo clot formation [12]. Taking a loss-of-function approach, we observed that TLR7^−/−^ septic platelets show better aggregation and adhesion compared to WT septic platelets. These findings indicate that TLR7 inflammatory signaling contributes to the development of platelet hyporeactivity in sepsis.

Platelet hyporeactivity in sepsis is characterized by a reduced responsiveness to agonists that normally trigger platelet aggregation. Yaguchi et al. [31] and Weiss et al. [34] both observed stable expression of major receptors in septic platelets including CD42a and CD42b (vWF), CD36 (collagen), PAR-1 (thrombin), and CD41/CD61 (fibrinogen, α2bβ3), and only a partial reduction in GPVI (collagen) expression compared to healthy platelets. Their findings strongly suggest that platelet hyporeactivity in sepsis is secondary to altered platelet bioenergetics and intracellular signaling, as opposed to decreases in the density of major receptors. For instance, septic platelets reached only 50% of the maximal aggregation achieved by healthy control platelets, and demonstrated reduced respiratory chain enzyme activity including NADH, and lower intracellular ADP and serotonin—major platelet granular components [35]. Although, these authors also point out that reduced granular components do not completely account for the impaired primary aggregation of platelets as initial aggregation precedes secretion in vitro, and indicates that other mechanisms are involved. Kao et al. determined that septic platelets show reduced adherence and spreading on fibrinogen compared to sham controls, and adding fibrinogen improved, but did not restore adhesion to control levels [36], suggesting that other factors affect platelet CD41/CD61 receptor binding and platelet hyporeactivity in sepsis. In vivo, the platelet GPVI receptor directly binds collagen to activate platelets and trigger calcium release from the dense tubular system (DTS) [37], while the GPIIbIIIa integrin receptor for fibrinogen shifts from low to high affinity during calcium-dependent platelet activation. Therefore, while septic platelets can bind collagen via the GPVI receptor, impaired calcium release can lead to reduce aggregation and possibly decreased fibrinogen binding and spreading.

We investigated whether septic platelet dysfunction is secondary to altered intracellular Ca^2+^ release. We observed that septic platelets have attenuated calcium flux in response to high-dose thrombin. If platelet receptor density is unchanged in sepsis, our findings instead indicate a receptor desensitization in which platelet responses, specifically calcium release from the dense tubular system, are blunted, and phenotypically observed as diminished platelet aggregation. A similar process is seen in platelets isolated from elderly patients in which calcium flux is only abnormal following treatment with a high concentration of agonists and is likely related to chronic receptor stimulation from low grade, but persistent thrombin generation [38]. Sepsis presents a similar process of persistent inflammation, thrombin generation, and platelet activation [2], leading to platelet receptor desensitization, as evidenced by the blunted calcium flux, and ultimately poor aggregation profiles. We further consider that calcium stores in septic platelets might be depleted or exhausted. However, our experimental results demonstrate that baseline calcium transients (baseline equal to the 60 s period prior to thrombin stimulation~0.1 µM) were similar across all mice and groups, suggesting that prior to stimulation there is no difference in basal calcium flux. After stimulation, cytosolic calcium levels increased in sham and septic samples, but over time we observed a difference in calcium oscillations, with sham platelets reaching peaks between 10 and 100 µM, while CLP platelets reached peaks consistently between 1 and 10 µM. This response range is consistent with other research, which has shown calcium concentrations in the platelet cytoplasm range between 0.025 and 0.1 µM, but within the DTS, storage concentrations can reach up to 500 µM in a resting platelet and be available for stimulated release [39,40]. Our findings therefore support the premise that persistent platelet activation in sepsis leads to platelet receptor desensitization, indicated by blunted calcium responses to agonists. Notably, TLR7^−/−^ septic platelets demonstrate preserved, and even enhanced total calcium release, in response to exogenous agonists. This observation provides a potential rationale for the preserved platelet aggregatory function seen with TLR7^−/−^ septic mice.

TLR7 recognizes single-stranded RNAs and has a nuanced role in polymicrobial sepsis. Although TLR7 signaling in platelet activation historically has been studied in viral diseases such as EMCV-1 [41], TLR7 has also been implicated in dysregulated and persistent inflammation in sepsis, secondary to the recognition of endogenous DAMPs, most notably ex-miRNAs. [13,14,18] Given the impact TLR7 deficiency has on platelet counts [12], activation [11], and function in sepsis (current data), accumulating evidence again points to a plasma component mediating these effects. Our previous work showed that septic plasma induced platelet activation in a partially TLR7-dependent manner [11], indicating the presence of a plasma mediator(s) that signals via TLR7. Potential candidates include ex-miRNAs, which are known to be elevated in septic plasma, make up 80% of plasma extracellular RNA [18,42], and are in part carried by EVs [43]. Prior work has shown that septic plasma EVs can induce inflammatory signaling in a partially TLR7-dependent manner via their miRNA content [21,22]. However, we found that septic plasma EVs induced PLA formation independent of TLR7. Given the heterogeneity of EV cargo and potential for synergistic signaling, these results were not unexpected. Additionally, prior work has shown plasma miRNA levels to be approximately 10-fold higher than the concentrations found in EVs [44], and therefore at the physiological dose applied in this study, they may not be potent enough to produce an effect in vitro. Therefore, our future studies may focus on the extraction and purification of endogenous plasma miRNAs from septic samples to determine their direct impact on platelet activation.

**Figure 6:**
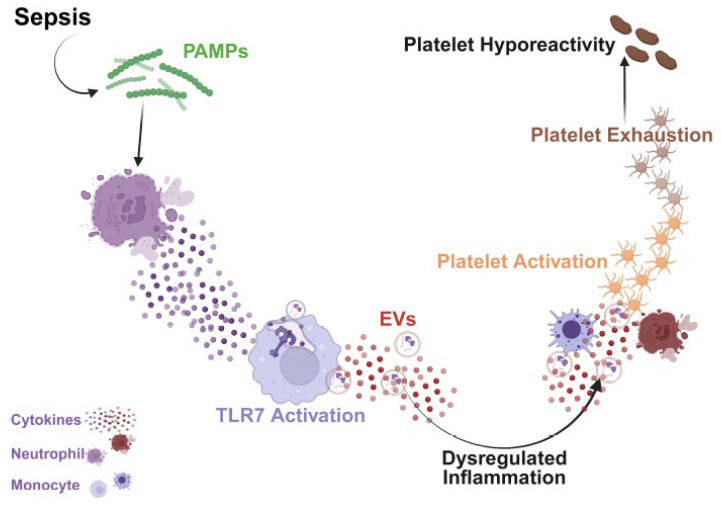
Working model of acquired platelet dysfunction in sepsis. Release of pathogen-associated molecules (PAMPs) in sepsis triggers immune cell activation and the release of cytokine and host-associated danger molecules, or DAMPs. Specific DAMPs can activate the innate immune receptor, Toll-like receptor 7 (TLR7). This signaling triggers further leukocyte activation, cytokine and DAMP release, including EVs, and perpetuates dysregulated host response and a highly proinflammatory environment that in turn leads to persistent platelet activation. Eventually, this chronic activation of circulating platelets leads to a state of exhaustion phenotypically manifested as decreased responsiveness to exogenous agonists, or hyporeactivity.

Although the biological character and function of plasma EVs in sepsis remain under investigation, previous work has shown differentiation in EV cargo proteins when comparing healthy control and sepsis samples. A study by Li et al. discovered that 99 EV cargo proteins were differentially expressed throughout the course of sepsis (sepsis→severe sepsis→septic shock) [45]. Further, analysis of the upregulated proteins revealed network interactions subcategorized into three main protein complex modules, including a module in which proteins were significantly enriched in inflammation and TLR-signaling, blood coagulation, and platelet degranulation processes. [45] Plasma EVs have heterogenous cargo based on cellular origin (e.g., ECs, leukocytes, platelets) and donor cell activation state, with potential enrichment of procoagulant and proinflammatory proteins in disease conditions. In fact, our recent publication demonstrates [46] that human septic EVs possess a proteome cargo highly enriched for EC activation, innate immune/inflammation, and coagulation signaling pathways. Building on this premise that EV cargo is modified during sepsis and is associated with disease severity—potentially altering EV activity—we silenced TLR7 in our sepsis model and determined the impact on septic EV biological function. Based on a report by Leopold et al., who demonstrated that COVID-19 plasma induces phosphatidylserine externalization in platelets [47], we incubated naïve platelets with either WT or TLR7^−/−^ septic plasma or their respective plasma EVs. Both WT and TLR7^−/−^ septic plasma induced comparable levels of platelet activation. However, TLR7^−/−^ septic EVs induced less platelet activation and PLA formation in whole blood compared to WT septic EVs. The findings indicate that TLR7 activation in sepsis drives inflammation, which alters the functional character of plasma EVs, promoting platelet activation and exhaustion (Figure 6).

Sepsis involves an initial hyperinflammatory phase marked by the activation of platelets, leukocytes, and endothelial cells, and is followed by an immunosuppressive state characterized by immune cell dysfunction and death [48]. Early platelet activation corresponds with inflammation, while late platelet dysfunction is associated with immunosuppression and reflects the severity of sepsis [49]. Similarly to platelets, changes in responsiveness among peripheral blood innate immune cells in sepsis have been previously reported. Shalova et al. found that while septic monocytes show preserved phagocytic ability, there is significant attenuation of inflammatory cytokine response and antigen presentation to LPS, indicating endotoxin tolerance [50]. Likewise, septic neutrophils have shown reduced chemokine expression and chemotaxis in response to immune stimulation with fMLP (N-formyl-methionyl-leucyl-phenylalanine) [51]. We too previously found septic-induced functional alterations in circulating neutrophils. We demonstrated that although total blood leukocyte counts dropped in both WT and TLR7^−/−^ septic mice, WT mice demonstrated reduced neutrophil migration to the peritoneum, a lower number of phagocytic neutrophils, and higher bacterial load in the blood compared to TLR7^−/−^ septic mice [15]. These findings support our current observations of septic-induced platelet dysfunction mediated by TLR7. Excessive inflammation in sepsis creates an environment for the overactivation and functional exhaustion of peripheral innate immune cells, and TLR7 deficiency appears to be protective in this regard.

### Limitations

Our study has the following limitations. Plasma EVs are derived from nearly all cell types and tissues, and in this study, we did not attempt to source the EVs, given that we wanted to determine a comprehensive effect of circulating septic plasma EVs in platelet activation. Further, we focused on whether there was a difference in the functionality of septic plasma EVs depending on the presence of TLR7. Therefore, we did not determine the subsequent impact on EV protein cargo, but this larger investigation is a part of our future work. Next, we observed similar levels of soluble PF4 at 4 h post-CLP in WT and TLR7^−/−^ mice, indicating a similar degree of early platelet activation. This is in contrast to our prior study, in which we observed attenuated levels of circulating activated PLA in TLR7^−/−^ mice 4 h post-CLP, indicating a reduction in the early interaction of activated platelets and leukocytes. [11] Possible reasons for this discrepancy may be due to the endpoint analyzed, soluble factor versus cell surface protein, and the significant sample variation we observed with the PF4 assay, limiting our ability to detect a difference due to inadequate sample size. Further, sex was considered as a biological variable in our studies; in our pilot studies utilizing female and male mice, global coagulation using viscoelastic testing following the CLP procedure was performed, but we noted that female mice demonstrated less perturbations in global coagulation dysfunction compared to males. Therefore, out of necessity for maintaining adequate severity to establish baseline coagulopathy and platelet dysfunction, we subsequently utilized male mice. Ketamine and xylazine were our primary anesthetics, and ketamine has been shown to impair platelet function. However, at standard recommended doses (80 mg/kg–110 mg/kg), clinically significant platelet dysfunction may be negligible. For instance, a study by Sashindranath et al. found that choice of anesthetic (ketamine and isoflurane) influenced in vivo blood flow velocity and volume in an arterial thrombosis model, but had no impact on ex vivo platelet aggregation in response to thrombin and ADP. [52] Finally, we only focused on platelet activation and PLA formation in response to septic EVs, but the impact on other platelet functions will be investigated in the future.

## 5. Conclusions

In summary, our study shows that sepsis is linked to altered platelet function, characterized by activated yet hyporeactive platelets with diminished calcium signaling. Our results suggest that modulating inflammatory signaling through the absence of TLR7 leads to a significant reversal of this phenotype, preserving platelet functionality. Additionally, the absence of TLR7 signaling resulted in reduced EV-mediated platelet activation, indicating a potential connection between upstream TLR7 signaling, inflammation, and downstream pathological EVs in sepsis. Future research will investigate changes in EC–platelet–monocyte interactions, septic EV function and cargo, and the impact of TLR7 deficiency, and whether our findings can be replicated with pharmacological inhibition of TLR7 in sepsis.

## Data Availability

All data generated or analyzed in this study are included in the published article and its supplementary information files.

## Supplementary Materials

The following supporting information can be downloaded at https://www.mdpi.com/article/10.3390/biom15111604/s1, Figure S1: Gating strategy for washed platelets. Figure S2: Gating strategy for whole blood. Figure S3: Baseline aggregation in WT and TLR7^−/−^ mice. Figure S4: Baseline calcium flux in WT and TLR7^−/−^ mice. Figure S5: Septic EVs stimulate platelet activation independent of TLR7.
