# Supplementary material for: Toll-like Receptor 7 Deficiency Attenuates Platelet Dysfunction in Sepsis
Source: Biomolecules. 2025 Nov 15;15(11):1604. doi: 10.3390/biom15111604 (PMC12650191; doi:10.3390/biom15111604)
Supplement: Supplementary file 1 [file biomolecules-15-01604-s001.zip › biomolecules-3922221-supplementary.pdf]

Supplementary Figure Legends

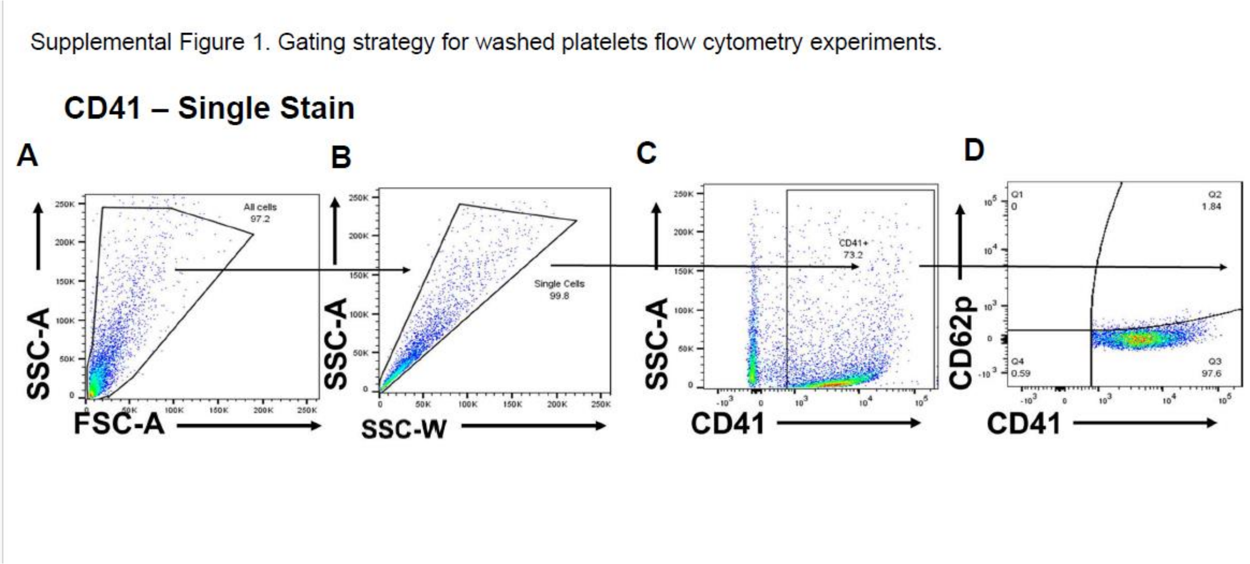

Supplementary Figure S1- Gating strategy for flow cytometry for washed platelets and activation. Platelets were gated on FSC-A and SSC-A (**A**) followed by gating for single cells (**B**) and then CD41^{+} cells (**C**). The percentage of CD62p^{+} cells of total CD41+ cells was calculated (**D**). CD41 single stain shown.

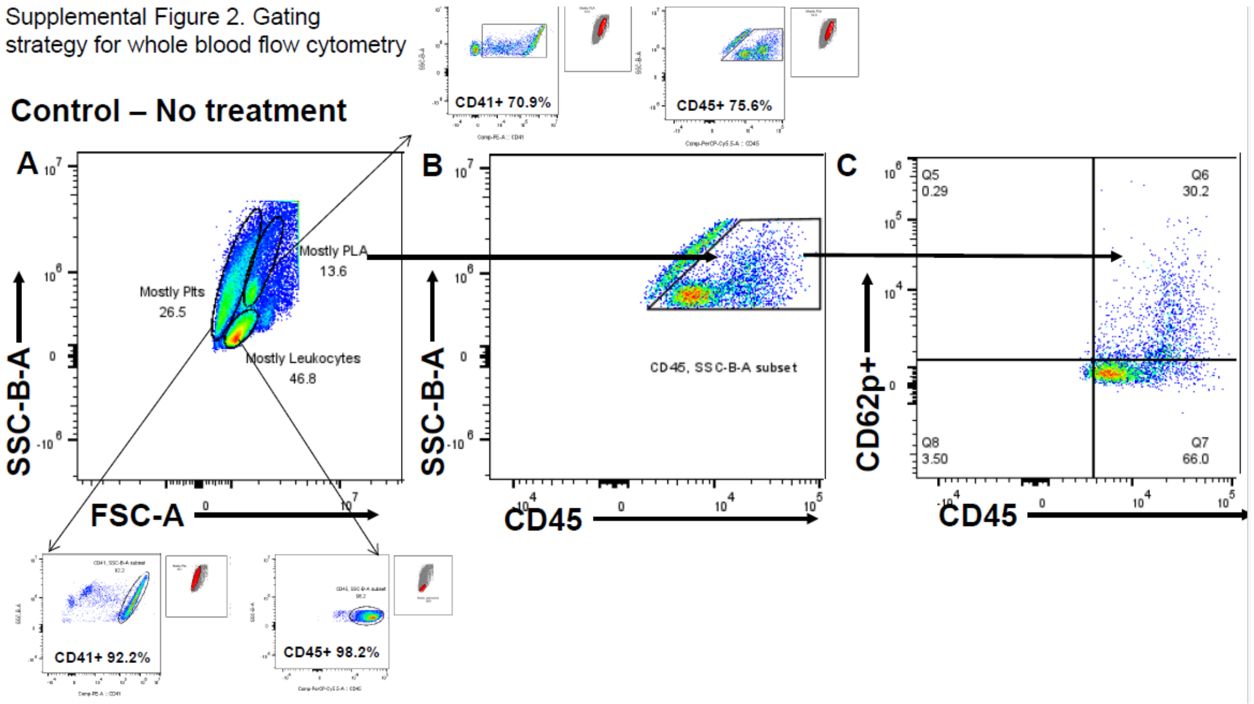

Supplementary Figure S2- Gating strategy for flow cytometry for platelet-leukocyte aggregate formation in whole blood. Based on FSC and SSC-B-A properties, we first determined platelet leukocyte and PLA population in lysed whole blood and confirmed using backgating strategies looking for platelet (CD41) and leukocyte (CD45) specific markers (**A**). Then from the PLA population we gated on CD45$^+$ (**B**) and then CD62p$^+$ (**C**) cells. The percentage of CD62+ cells out of PLA population was quantified. Small boxes represent backgating results. Control sample shown.

Supplemental Figure 3. Baseline aggregation in WT and TLR7$^{-/-}$ sham mice.

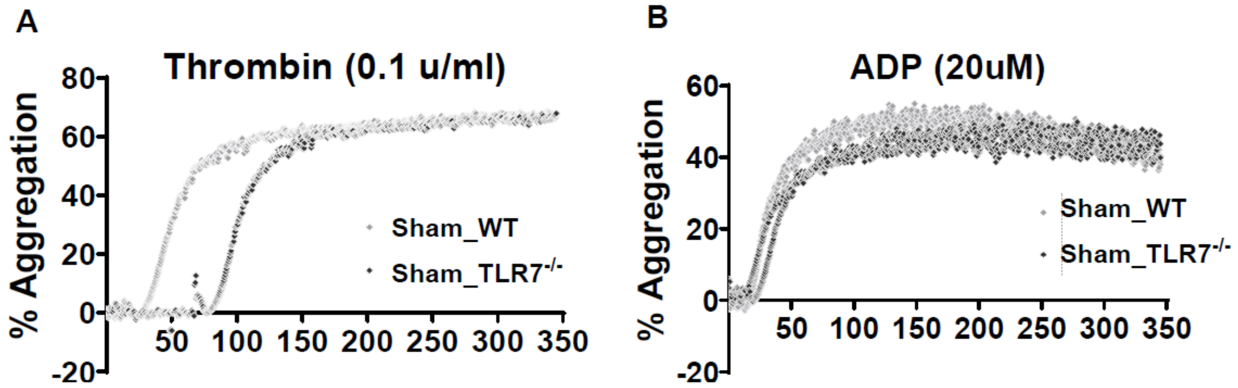

Supplementary Figure S3- Baseline aggregation in WT and TLR7$^{-/-}$ sham mice. We utilized whole body TLR7 knockout mice and therefore established baseline signals in both WT and TLR7$^{-/-}$ sham mice. Figure **A** and **B** demonstrate no difference in maximal aggregation between WT and TLR7$^{-/-}$ sham mice to thrombin or ADP.

Supplemental Figure 4. Baseline Calcium Flux in Naive WT and TLR7$^{-/-}$ Platelets

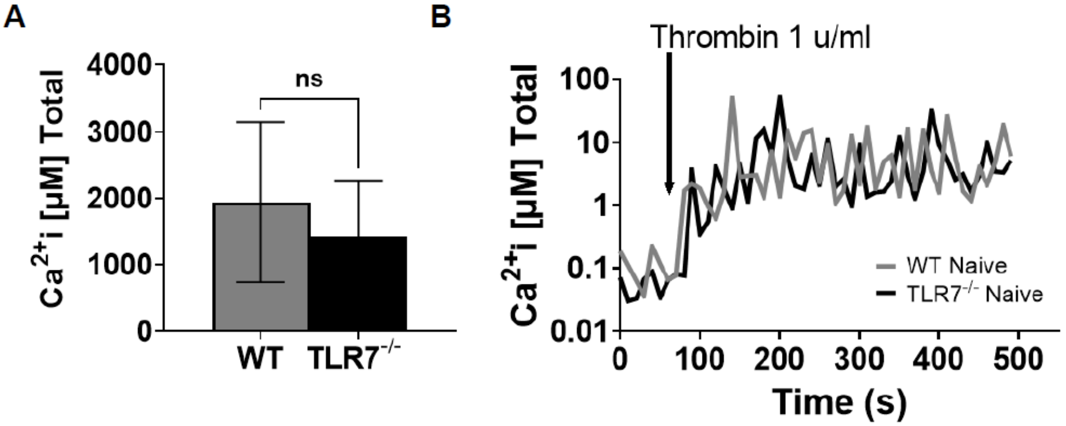

Supplementary Figure S4- Baseline calcium transients in WT and TLR7$^{-/-}$ naive mice. We utilized whole body TLR7 knockout mice and therefore established baseline calcium transients in both WT and TLR7$^{-/-}$ naive mice. Figure **A** and **B** demonstrate no difference in response to thrombin (1 u/ml) in terms of total [Ca2+]i between WT and TLR7$^{-/-}$ naive mice.

Supplemental Figure 5. Septic EVs stimulate platelet activation independent of TLR7.

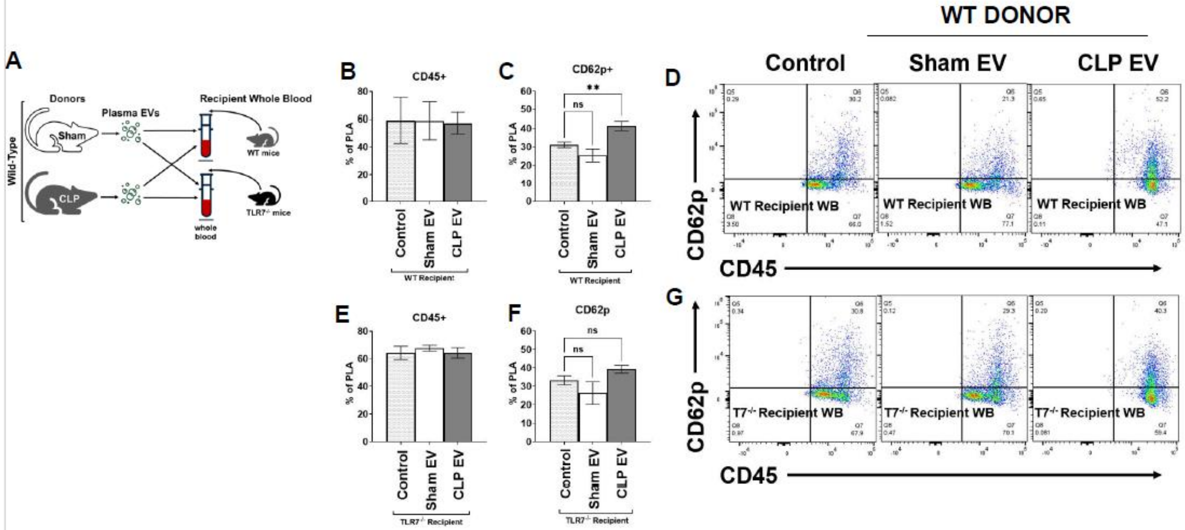

Supplementary Figure S5- Septic EVs stimulate downstream platelet activation independent of TLR7. WT mice underwent sham or CLP procedure and at 24H whole blood was collected and EVs isolated from plasma. EVs were incubated with whole blood from naïve WT or TLR7$^{-/-}$ mice (**A**) and activated PLAs quantified (CD62p^{+}CD45^{+}/CD45^{+} PLAx100%) as before. EV dose was 2.5x10^{10} EVs/ml for all experiments. WT CLP EVs induced more activated PLA compared to control in WT recipient whole blood (**B-D**), while in TLR7$^{-/-}$ recipient whole blood, WT CLP EVs triggered a similar increase in activated PLA *vs* controls, although this difference did not reach statistical significance (**E-G**).
